# Antimicrobial peptide cWFW kills by combining lipid phase separation with autolysis

**DOI:** 10.1038/srep44332

**Published:** 2017-03-09

**Authors:** Kathi Scheinpflug, Michaela Wenzel, Oxana Krylova, Julia E. Bandow, Margitta Dathe, Henrik Strahl

**Affiliations:** 1 Peptide-Lipid Interaction, Leibniz-Institut für Molekulare Pharmakologie (FMP), Berlin, Germany; 2 Applied Microbiology, Ruhr University Bochum, Bochum, Germany; 3 Centre for Bacterial Cell Biology, Institute for Cell and Molecular Biosciences, Newcastle, University, Newcastle upon Tyne, United Kingdom

## Abstract

The synthetic cyclic hexapeptide cWFW (cyclo(RRRWFW)) has a rapid bactericidal activity against both Gram-positive and Gram-negative bacteria. Its detailed mode of action has, however, remained elusive. In contrast to most antimicrobial peptides, cWFW neither permeabilizes the membrane nor translocates to the cytoplasm. Using a combination of proteome analysis, fluorescence microscopy, and membrane analysis we show that cWFW instead triggers a rapid reduction of membrane fluidity both in live *Bacillus subtilis* cells and in model membranes. This immediate activity is accompanied by formation of distinct membrane domains which differ in local membrane fluidity, and which severely disrupts membrane protein organisation by segregating peripheral and integral proteins into domains of different rigidity. These major membrane disturbances cause specific inhibition of cell wall synthesis, and trigger autolysis. This novel antibacterial mode of action holds a low risk to induce bacterial resistance, and provides valuable information for the design of new synthetic antimicrobial peptides.

Antimicrobial peptides (AMPs) have been in the focus of research attention as a promising alternative for conventional antibiotics^1^. The subclass of arginine- and tryptophan-rich peptides, with cationic charge and hydrophobic residues, provides the ideal structural prerequisite for interaction with the negatively charged bacterial membranes^2^. To elucidate optimal structure-activity properties we screened a library of small synthetic peptides derived from the active motif of human antimicrobial peptide lactoferricin^3^. This analysis identified the cyclic hexapeptide cWFW (cyclo-RRRWFW) as the most potent candidate. It turned out that this peptide is active against both Gram-positive and Gram-negative bacteria at low micromolar concentrations, and displays strong selectivity, i.e. it is not toxic towards human cells^4^. The antibacterial activity of cWFW is further characterised by a rapid killing kinetic and a bactericidal effect at the minimal inhibitory concentration^5^. To develop cWFW as a novel, highly efficient antibiotic lead compound, we now need a precise knowledge of its mechanism of action.

Earlier studies have suggested that the cytoplasmic membrane is the primary peptide target but it is unknown how this interaction kills bacteria^5^,^6^. In contrast to most AMPs, however, cWFW does not permeabilize the cell membrane to large molecules^5^,^6^. As first postulated by Epand and Epand^7^, short cationic AMPs might, as an alternative mechanism, induce clustering of anionic lipids within the bacterial membrane due to electrostatic interactions^8^. This effect, which is highly dependent on the phospholipid composition and requires the presence of both negatively charged and zwitterionic lipid species, could later be confirmed for small cyclic hexapeptides including cWFW *in vitro*^9^,^10^. Whether this clustering indeed takes place *in vivo*, and whether it contributes to the antibacterial activity of cWFW has so far remained unanswered.

In this manuscript, we now present a comprehensive mode of action analysis demonstrating that cWFW, uniquely for a cationic antimicrobial peptide, neither depolarises the membrane nor otherwise adversely affects the energy state of the cell. Instead, cWFW strongly reduces membrane fluidity both *in vivo* and *in vitro*, and triggers a large scale phase-separation of the cytoplasmic membrane. In contrast to the previously suggested models, the cWFW-triggered lipid phase separation is independent of both the negatively charged phospholipid cardiolipin and the zwitterionic phospholipid phosphatidyl ethanolamine, and thus represents a novel activity for a membrane-targeting peptide. Moreover, the *in vivo* domain formation causes a striking segregation of peripheral and integral membrane proteins into separate membrane areas. This strong disturbance of membrane organisation inhibits cell wall synthesis and triggers autolysis. We consider these effects as a novel mechanism of action for antimicrobial peptides, and discuss the implication on antimicrobial resistance.

## Results

### Analysis of the cWFW-triggered cellular stress response

**Figure 1:**
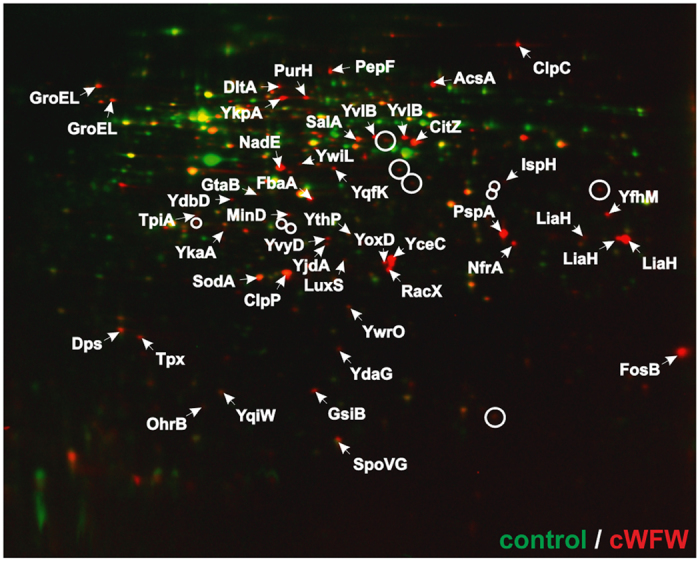
Cytosolic proteome response profile of *B. subtilis* to cWFW. Autoradiographs of antibiotic-treated cells (red) were overlaid with those of untreated controls (green). Upregulated proteins in response to cWFW treatment (8 μM) appear red, down-regulated proteins green. Proteins synthesised at equal rates appear yellow. Proteins upregulated more than 2-fold in three biological replicates were defined as marker proteins and identified by mass spectrometry. Unidentified marker proteins are indicated by circles. See supplementary Figure 1a for the growth inhibition of *B. subtilis* observed in BMM with different cWFW concentrations. Strain used: *B. subtilis* 168/DSM 402.

To identify which cellular processes are disturbed by cWFW, we performed proteomic profiling of the Gram-positive model organism *B. subtilis* (Fig. 1). This technique combines radioactive pulse-labeling of newly synthesised proteins with two-dimensional SDS PAGE, thus providing insight into the acute antibiotic stress response^11^. The proteomic stress response profile, consisting of marker proteins upregulated after antibiotic addition, is highly specific for the imposed stress and thus indicative of the antibiotic’s mechanism of action^11^.

In agreement with the cytoplasmic membrane as the likely cellular target^6^, cWFW elicited a typical cell envelope stress response characterised by upregulation of proteins controlled by the extracytoplasmic function (ECF) sigma factors SigM, SigW and SigX (Table 1). In addition, marker proteins indicative of membrane damage (phage shock protein PspA and NAD synthase NadE) and impairment of membrane-bound steps of cell wall synthesis (PspA homologue LiaH and the Sigma M-controlled protein YpuA) were upregulated. Further upregulated proteins point towards remodeling of the cell wall (RacX, DltA) and membrane (YjdA, YoxD, IspH), accompanied by changes in central carbon metabolism (TpiA, FbaA, CitZ) and activation of the SigB-dependent general stress response.

When comparing the cWFW-triggered stress response to a proteome response library^12^ we found the highest overlap with the antimicrobial peptide MP196, but also a striking similarity with gramicidin S and valinomycin (Supplementary Table 1). The linear hexapeptide MP196 de-energises the cell by inhibition of respiration^13^ whereas the cyclodecapeptide gramicidin S dissipates membrane potential via transient ion conductance events^14^. Membrane depolarisation caused by the ion carrier valinomycin is a direct result of specific potassium transport^15^. Similarities in the proteomic response elicited by compounds which directly interfere with cell wall synthesis such as vancomycin and mersacidin, or other specific ionophores such as gramicidin A or ionomycin were comparably low (Supplementary Table 1).

Together, these results argue against a specific proteinaceous target for cWFW. Instead, cWFW is more likely to target the cytoplasmic membrane and to cause extensive interference with membrane-associated processes including energy metabolism, cell wall synthesis, and membrane homeostasis. The following paragraphs focus on how cWFW affects these different aspects of *B. subtilis* physiology.

### Membrane permeability and cell energy state

Earlier investigations have suggested that cWFW does not permeabilize the cytoplasmic membrane of *E. coli*, or form pores in model membranes^5^,^6^. However, the induction of the general SigB-dependent stress response and the similarity of the proteomic response triggered by MP196 and gramicidin S suggests that cWFW might nevertheless cause energy starvation linked to membrane depolarisation in *B. subtilis*.

**Figure 2:**
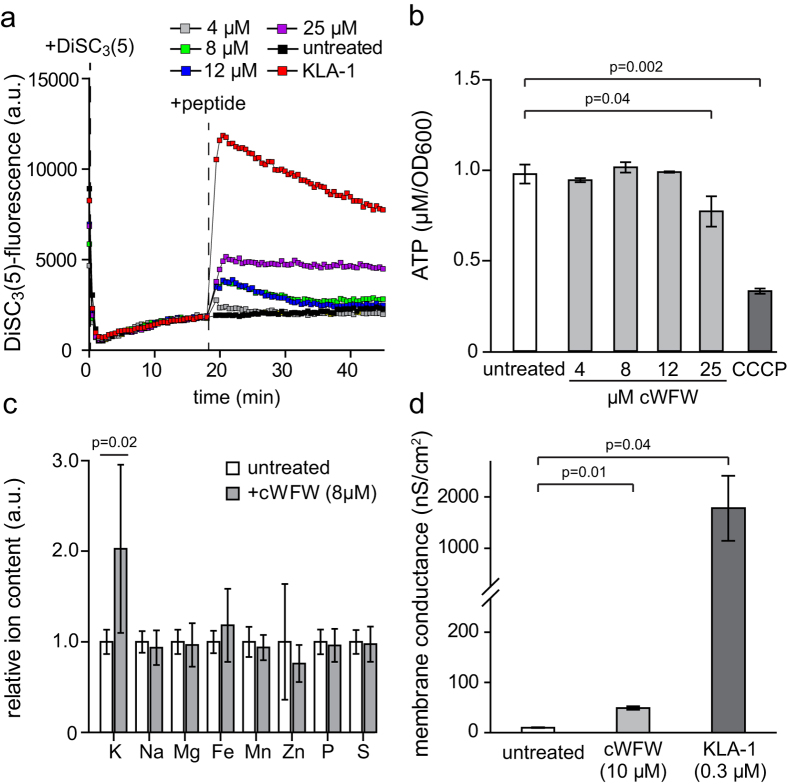
Impact of cWFW on cell energy state and membrane permeability. (**a**) Membrane potential levels of *B. subtili*s upon addition of different cWFW concentrations were measured using fluorescent voltage-sensitive dye DiSC_3_(5). For positive control, cells were depolarised by addition of the helical pore-forming antimicrobial peptide KLA-1 (40 μM). The time points of DiSC_3_(5) and peptide additions are highlighted with dashed lines. See supplementary Figure 1b for growth inhibition at identical cWFW-concentrations and cell densities. The graph depicts a representative measurement of three independent replicates. (**b**) ATP levels in *B. subtilis* after 20 min incubation with different cWFW concentrations were measured using a Luciferase-based luminescence assay. For positive control, cells were incubated with the proton ionophore CCCP (100 μM). Cell densities are comparable to the data shown in panel A and supplementary Figure 1b. The diagram depicts the average and standard deviation values of three independent replicates. No significant changes (p ≥ 0.05) were observed for samples treated with 4, 8, and 12 μM cWFW. (**c**) Changes in relative ion content of *B. subtili*s upon 15 min incubation with 8 μM cWFW were determined using inductively-coupled plasma optical emission spectroscopy (ICP-OES). Phosphorus, mainly prevalent in DNA-bound form, served as internal control for cell mass. The diagram depicts the average and standard deviation values of three independent measurements. No significant changes (p ≥ 0.05) were observed ions other than K^+^. See supplementary Figure S1a for the growth inhibition of *B. subtilis* observed in BMM with different cWFW concentrations. (**d**) Conductivity measurements on planar lipid membranes formed of *E. coli* lipid extract upon addition of 10 μM cWFW. The pore-forming helical peptide KLA-1 (0.3 μM) served as positive control. The diagram depicts the average and standard error of two independent measurements. The statistical significances were calculated using unpaired (panels b/d) and paired (panel c) two-tailed Student t test. Strains used: (a/b) *B. subtilis* 168, (**c**) *B. subtilis* 168/DSM 402.

To test this possibility, we first analysed the cellular membrane potential with the voltage-sensitive dye DiSC_3_(5)^16^. As shown in Fig. 2a, the fluorescence intensity of DiSC_3_(5)-stained cell suspension was slightly increased at growth-inhibitory concentrations (8 μM) and above (Supplementary Figure 1). This indicates a detectable degree of membrane depolarisation caused by cWFW but the observed effect was very low compared to that of the positive control KLA-1, which is a pore-forming peptide fully dissipating the membrane potential^17^. In addition, cWFW-induced depolarisation was only transient at growth-inhibitory concentrations (8–12 μM) and the membrane potential was restored almost to control levels within 10 min (Fig. 2a). In agreement with these measurements, a minor reduction of cellular ATP levels was observed only at elevated concentrations of cWFW (Fig. 2b).

To verify these findings with independent assays, we next analysed cWFW-triggered changes in cellular ion content, and performed conductivity measurements on bacterial model membranes. Consistent with the lack of significant membrane depolarisation, no leakage of cellular ions was observed *in vivo* (Fig. 2c). In contrast, an accumulation of K^+^ upon addition of cWFW was observed. At last, only a minor ion flux was induced in model bilayers compared to the pore-forming peptide KLA-1 (Fig. 2d).

Together, these analyses demonstrate that cWFW neither permeabilizes the cytoplasmic membrane nor acts as a carrier for the analysed ion species. cWFW thus exhibits a mode of action which clearly sets it apart from typical membrane targeting antimicrobial peptides which either form ion-conducting pores, or otherwise interfere with the membrane diffusion barrier function and cell energy state.

### cWFW interferes with membrane homeostasis by reducing membrane fluidity

Although the membrane barrier function is not significantly impaired, there is convincing evidence that cWFW can directly influence lipid bilayers upon binding^10^,^18^. Therefore, we focussed on analysing two plausible effects through which cWFW could interfere with the bacterial cytoplasmic membrane: (*i*) disturbance of membrane homeostasis and (*ii*) lipid domain formation.

**Figure 3:**
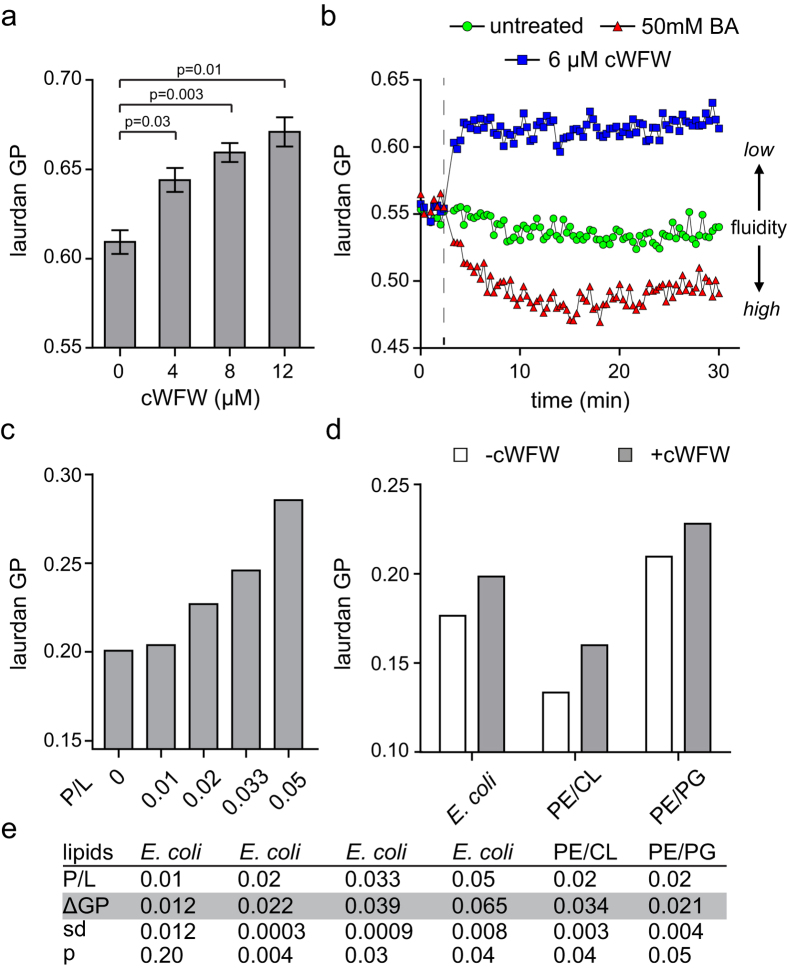
cWFW reduces membrane fluidity *in vivo* and *in vitro.* (**a**) The fluidity of the cytoplasmic membrane was measured for *B. subtilis* cells upon 10 min incubation with increasing concentrations of cWFW using the fluidity-sensitive fluorescent dye laurdan. Please note that high laurdan generalised polarisation (GP) correlates with low membrane fluidity. The diagram depicts the average and standard deviation of three replicate measurements. (**b**) Time-resolved laurdan generalised polarisation (GP) was measured upon addition of 6 μM cWFW. As a positive control, 50 mM of the membrane fluidiser benzyl alcohol (BA) was added to a replicate sample. The time point of addition is indicated with a dashed line. The graph depicts a representative measurement of three independent replicates. (**c**) Laurdan GP was measured for large unilamellar vesicles (LUVs) formed of *E. coli* polar lipid extract in the presence of increasing concentrations of cWFW. The peptide-to-lipid molar ratios (P/L) are indicated below the graph. See supplementary Figure S3b for examples of the recorded spectra. (**d**) Laurdan GP in the presence and absence of cWFW (P/L = 0.02) was measured for LUVs with varying lipid compositions (lipid molar ratios: PE/CL 87.5/12.5, PE/PG 75/25). See supplementary Figure 3c–f for recorded spectra. PE: palmitoyl-oleoyl-phosphatidylethanolamine, PG: palmitoyl-oleoyl-phosphatidylglycerol, CL: cardiolipin and *E. coli: E. coli* polar lipid extract. (**e**) Average and standard deviation of cWFW-induced changes in laurdan GP (ΔGP) are shown for the different lipid compositions and peptide-to-lipid molar ratios (P/L) from two independent experiments. The statistical significances were calculated using unpaired (panels a) and paired (panel e) two-tailed Student t test.

Biological membranes are maintained in an appropriate state of fluidity which optimally promotes the activity and diffusion of membrane proteins while maintaining sufficiently low passive permeability^19^. Antimicrobial peptide-triggered changes in fluidity have been observed in model membranes systems *in vitro*^20^,^21^, and very recently for the lipopeptide daptomycin *in vivo*^22^. To test whether cWFW can disturb the fluidity of the cytoplasmic membrane, we used the fluidity-sensitive fluorescent dye laurdan. Indeed, a clear concentration-dependent increase in laurdan generalised polarisation (GP) was observed upon incubation of *B. subtilis* with cWFW (Fig. 3a). This indicates a substantial reduction of membrane fluidity. Crucially, minor changes were already observed at concentrations which are growth-limiting but not sufficient to fully abolish growth (Supplementary Figure 1).

*B. subtilis* can adjust the membrane fatty acid composition and thus membrane fluidity upon stress^23^. Unsurprisingly, the cWFW-dependent membrane disturbances also trigger an adaptive response on the level of membrane fatty acid composition. (Supplementary Figure 2). To distinguish between a direct activity of cWFW and a potential cellular adaptation process, we measured time-resolved laurdan GP upon addition of cWFW (Fig. 3b). It turned out that the reduction of membrane fluidity occurs very rapidly upon addition and reaches a steady state within only 2 min, thus suggesting that observed membrane rigidification is a direct consequence of cWFW-membrane interaction.

To verify that the reduction of membrane fluidity is indeed direct activity of cWFW and to gain insight into a possible lipid-specificity of this phenomenon, we analysed the ability of cWFW to reduce membrane fluidity in an *in vitro* model system using large unilamellar vesicles (LUVs) composed of bacterial membrane lipids. A distinct concentration-dependent reduction of membrane fluidity was observed with liposomes formed from *E. coli* polar lipid extracts (Fig. 3c, Supplementary Figure 3b). This confirms that membrane rigidification is indeed a direct consequence of cWFW intercalation into the lipid bilayer. We next analysed whether the ability of cWFW to reduce membrane fluidity requires a complex fatty acid composition found in biological membranes, or relies on the presence of specific phospholipid head group species. For this aim, we repeated the experiments with liposomes composed of binary mixtures of POPE/POPG and POPE/CL, respectively. A clear reduction of membrane fluidity was observed in both cases, indicating that the peptide activity is independent of the investigated composition of POPE-based lipid mixtures which mimic bacterial membranes (Fig. 3d and e). However, we did observe a slightly stronger reduction of fluidity in liposomes containing cardiolipin, a result consistent with the preferred partitioning of cWFW into cardiolipin-containing POPE membranes^6^.

In summary, the intercalation of cWFW into lipid bilayers results in a rapid reduction of membrane fluidity both on the cellular level and in model lipid bilayers. For the investigated bilayer systems, the ability to reduce membrane fluidity is independent of specific fatty acids or lipid head groups.

### cWFW triggers a novel type of lipid domain formation *in vivo*

**Figure 4:**
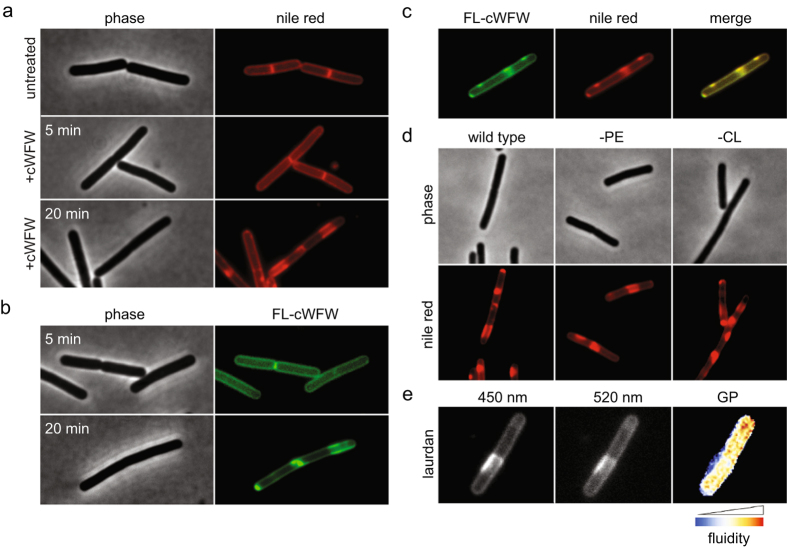
cWFW triggers large-scale lipid domain formation *in vivo.* (**a**) Phase contrast and fluorescence images of *B. subtilis* cells stained with fluorescent membrane dye nile red before addition (upper panels) and after 5 min (middle panels) and 20 min (lower panels) incubation with cWFW (12 μM). (**b**) Phase contrast and fluorescence images of *B. subtilis* cells stained with a 1:5 mix of NBD-labelled (FL-cWFW) and unlabelled cWFW (combined concentration of 12 μM) after 5 min (upper panels) and 20 min (lower panels) incubation. (**c**) Fluorescence images of *B. subtilis* cells stained with NBD-labelled peptide (FL-cWFW; left panel) and nile red (middle panel) upon 20 min incubation. The right panel depicts a colour overlay of the images shown in left and middle panels. (**d**) Phase contrast and fluorescence images of *B. subtilis* cells stained with fluorescent membrane dye nile red after 20 min incubation with cWFW (12 μM). Depicted are wild type cells (left panels), cells deficient for phosphatidylethanolamine (-PE, middle panels), and cells deficient for cardiolipin (-CL, right panels). (**e**) Fluorescence images of *B. subtilis* cell stained with laurdan upon 20 min incubation with cWFW (12 μM). Depicted is the laurdan emission at 450 nm (left panel), 520 nm (middle panel) and a colour-coded laurdan GP map calculated from the images shown in left and middle panels, respectively. Strains used: (**a**–**e**) *B. subtilis* 168 (wild type), (**d**) *B. subtilis* HB5343 (Δ*psd*, PE-deficient) and *B. subtilis* SDB206 (Δ*clsA*, Δ*clsB*, Δ*ywiE*, CL-deficient).

The reduction of membrane fluidity upon addition of cWFW can either be caused by a uniform effect on the cytoplasmic membrane or by formation of specific domains which differ in fluidity. To address this question, we analysed cells stained with the fluidity-sensitive uncharged membrane dye nile red^24^,^25^. Upon addition of cWFW, the fluorescent membrane stain initially remained homogeneous indicating that the reduction of membrane fluidity extends to the entire cytoplasmic membrane (Fig. 4a). However, after a prolonged incubation, a striking separation of the membrane into two distinct areas was observed (Fig. 4a). This lipid domain formation was also reflected in the distribution of fluorescently labelled cWFW (Fig. 4b and c).

**Figure 5:**
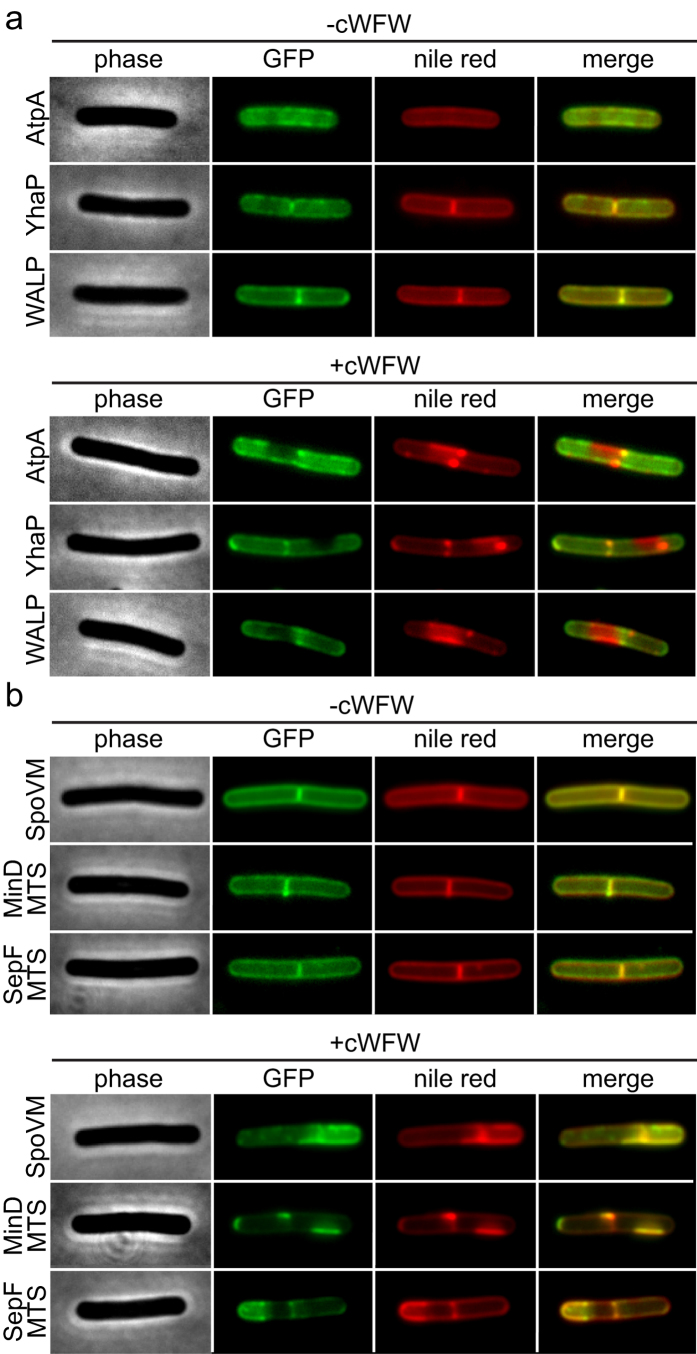
cWFW-triggered lipid domain formation segregates membrane proteins. (**a**) Phase contrast, GFP-fluorescence, nile red-fluorescence and fluorescent colour overlays are depicted for cells expressing different integral membrane proteins in the absence (upper panels) and presence (lower panels) of cWFW (20 min incubation with 12 μM). (**b**) Comparable images are depicted for cells expressing different peripheral membrane proteins. Strains used: (**a**) *B. subtilis* BS23 (AtpA-GFP), *B. subtilis* HS41 (YhaP-GFP), *B. subtilis* HS64 (WALP23-GFP), (**b**) *B. subtilis* KR318 (SpoVM-GFP), *B. subtilis* HS65 (GFP-MinD_MTS_) and *B. subtilis* HS208 (SepF_MTS_-GFP).

We have previously shown that cWFW treatment results in lipid demixing of PE/PG bilayers *in vitro*^9^,^10^. Moreover, PE and cardiolipin have been reported to form lipid domains in *B. subtilis* membranes^26^. The observed domains could therefore indicate aberrant clustering of these specific lipid species. To test this hypothesis, we repeated nile red staining with cells incapable of producing the zwitterionic PE or the negatively charged cardiolipin, respectively. To our surprise, the absence of neither of these major phospholipid species had an influence on the domain formation (Fig. 4d) or on the MIC of cWFW (Supplementary Table 4). Hence, the observed *in vivo* phase separation is not determined by clustering of PE or CL, or by the demixing of PE and negatively charged phospholipids. Instead, a microscopic analysis of laurdan GP revealed a clear difference in the local fluidity of the observed domains (Fig. 5e). The cWFW-triggered lipid domain formation thus exhibits characteristics reminiscent of membrane phase separation driven by changes in the physical state of the membrane, rather than by enrichment of specific lipid species.

### Impact of cWFW-induced lipid domain formation on protein localisation

The massive cWFW-triggered phase separation of the cytoplasmic membrane is prone to influence the distribution of membrane proteins. To analyse the general impact of cWFW on membrane protein localisation, we followed three dispersedly localised integral membrane proteins^24^. The chosen native proteins were YhaP, a Na^+^ efflux pump, and AtpA, a subunit of the F_1_F_o_ ATP synthase. The selection was supplemented with WALP23, an artificial model transmembrane helix^27^. For comparison, we investigated three dispersedly localised peripheral membrane proteins which bind the membrane via an amphipathic helix. These proteins were the native sporulation protein SpoVM^28^ and two semi-synthetic proteins composed of GFP fused with the amphipathic helices of the cell division protein SepF and the cell division placement protein MinD, respectively^29^. As shown in Fig. 5, all six tested membrane proteins were clearly delocalised upon cWFW treatment, thus demonstrating that the lipid domain formation indeed strongly affects membrane protein localisation (Fig. 5a). Surprisingly, whereas the integral membrane proteins were excluded from peptide-enriched domains, the analysed peripheral membrane proteins were accumulated in exactly these regions (Fig. 5b).

Taken together, the cWFW-triggered membrane phase-separation not only severely disrupts the overall membrane protein organisation, but also segregates membrane proteins into separate areas based on the type of membrane anchor utilised. This effect will inevitably cause major disturbances in membrane-associated cellular processes, and provides an explanation for the multitude of inhibitory effects observed in the proteome analysis.

### Impact of protein delocalisation on inhibition of cell wall synthesis

**Figure 6:**
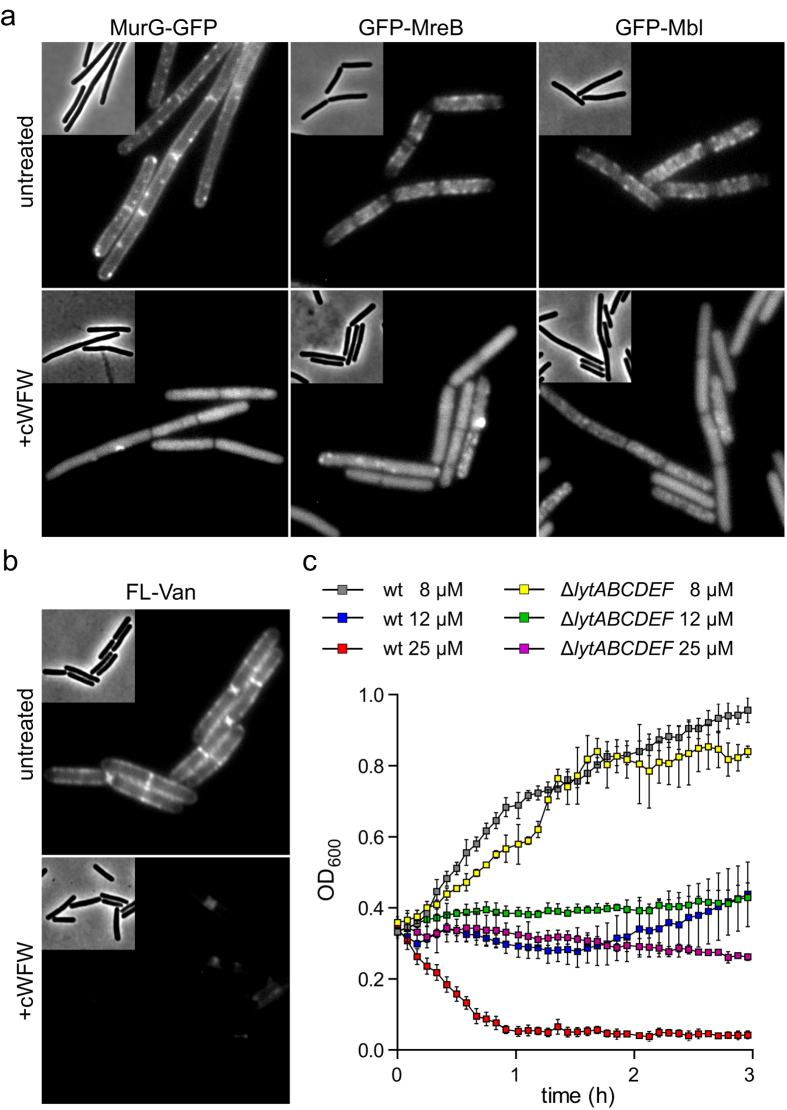
cWFW inhibits cell wall synthesis and triggers autolysis. (**a**) Phase contrast and fluorescence images of *B. subtilis* cells expressing MurG-GFP, GFP MreB and GFP-Mbl are depicted in the absence (upper panels) and presence (lower panels) of cWFW (20 min incubation with 12 μM). (**b**) Phase contrast and fluorescence images of *B. subtilis* wild type cells stained with fluorescent vancomycin (FL-Van) in the absence (upper panel) and presence (lower panel) of cWFW (20 min incubation with 12 μM). (**c**) Changes in optical density of *B. subtilis* wild type cells (wt), and cells deficient for the autolytic enzymes LytCDEF upon incubation with different concentrations of cWFW. The diagram depicts the average and standard deviation of three replicate cultures. Please note that the *lytABC* operon encodes the amidase LytC and accessory proteins LytAB which are involved in regulation and secretion of LytC, respectively. Strains used: (**a**) *B. subtilis* 168, *B. subtilis* KS69 (GFP-MreB), *B. subtilis* KS70 (GFP-Mbl), and *B. subtilis* TNVS175 (MurG-GFP), (**b**) *B. subtilis* 168, and (**c**) *B. subtilis* 168 (wt) and *B. subtilis* KS19 (Δ*lytABCDEF*).

Having identified the reduction of membrane fluidity and membrane phase separation as the mechanistic basis of the antimicrobial activity of cWFW, we further investigated how this leads to the observed rapid growth-inhibition of fully energised cells. *B. subtilis* grows by integrating two main processes: localised cell wall synthesis expanding the cell envelope and cell division separating the daughter cells. The proteome profile indicated that cWFW triggers a stress-response associated with inhibition of cell wall precursor lipid II biosynthesis, an essential process for both cell elongation and division. The observed inhibition of cell growth could therefore be caused by interference with cell wall synthesis. Two central membrane associated players in this process are the glycosyltransferase MurG, which catalyses the last step of lipid II synthesis^30^, and bacterial actin homologs of the MreB-type, which govern the correct cellular positioning of the lateral cell wall synthetic machinery^31^. In agreement with the stress response indicating disturbance in lipid II synthesis, cWFW triggered a release of both MurG and MreB-homologs from the membrane (Fig. 6a). To support this finding, we analysed whether the bacteria are able to synthesise lipid II in the presence of cWFW by staining the cells with a fluorescent derivative of the lipid II-binding antibiotic vancomycin (FL-Van). In *B. subtilis*, a high carboxypeptidase activity of PBP5 removes the vancomycin target D-Ala-D-Ala from matured cell wall. As a consequence, FL-Van binding is directly associated with lipid II and newly synthetised cell wall^32^. Indeed, a strong decrease of FL-Van staining was observed in cWFW-treated cells indicating significantly reduced amounts of lipid II (Fig. 6b). Together these findings provide strong evidence that cell wall synthesis is efficiently inhibited by cWFW.

### Lipid domain formation triggers cell wall autolysis

Addition of elevated concentrations of cWFW not only inhibits growth but also induces cell lysis (Supplementary Figure 1, Supplementary Movie 1). This seemingly contradicts our results showing that the membrane integrity is not significantly compromised. There is, however, another possibility to explain the observed lysis. In growing cells the cell wall sacculus needs to expand by incorporation of new cell wall material. This process requires a finely tuned balance between cell wall synthesis and degradation of the existing cell wall by autolytic enzymes while continuously maintaining the crucial function as a support for turgor pressure^33^. Upon disturbance of the cell wall synthesis, the cytoplasmic membrane can become insufficiently supported against the large pressure difference between the interior and the exterior of the cell. The resulting cell lysis is classically observed with antibiotics which, unlike cWFW, directly target the cell wall synthetic machinery^34^. However, the MreB-cytoskeleton is also involved in the regulation of autolytic enzyme activities^35^. The release of MreB and Mbl from the membrane could thus disturb the essential tight control of these potentially destructive cell wall degrading enzymes. To test this hypothesis, we analysed the bacteriolytic activity of cWFW in the absence of major autolysins LytCDEF. Indeed, the lysis triggered by cWFW was fully abolished in this strain background although the cells were still efficiently growth-inhibited (Fig. 6c). These results confirm that the lytic activity of cWFW is not due to disintegration of the cell membrane. Rather, cWFW causes weakening of the cell wall sacculus mediated by the cells’ own autolytic enzymes. The bacteriolytic property of cWFW is thus a consequence of triggered autolysis.

## Discussion

In this study, we have analysed the mode of action of the small cyclic antimicrobial membrane-targeting peptide cWFW. We provide evidence that cWFW exhibits a complex mode of action which encompasses both the cytoplasmic membrane and the cell wall of *B. subtilis*. Upon integration into the membrane cWFW triggers a rapid reduction of membrane fluidity followed by lipid phase separation process. This domain formation not only disturbs the lipid matrix, but also causes a striking segregation of integral and peripheral membrane proteins into different membrane areas. Changes in the physical state and the composition of the immediate surrounding lipid bilayer can have a profound effect on membrane protein structure and function^36^,^37^,^38^. As a consequence, the cWFW-induced domain formation can both physically separate proteins which require close proximity for interaction or which catalyse successive steps in a biosynthetic pathway, and impair protein functionality due to conformational changes. The combined disturbances in membrane fluidity and protein localisation explain the broad cellular response to cWFW observed in the proteome analysis. In addition to causing general disarray of membrane-associated processes, cWFW inhibits cell wall synthesis by dissociating the actin homologs MreB and Mbl, and the lipid II synthesis protein MurG from the cytoplasmic membrane. In agreement with cell wall disturbance, the cell lysis triggered by cWFW is a consequence of misregulation of cell wall autolytic enzymes rather than membrane disruption. Therefore, the antibacterial mode of action of this small cyclic cationic peptide is a combination of strong interference with general membrane organisation accompanied by an inhibition of cell wall synthesis and triggered autolysis.

Cationic antimicrobial peptides are generally assumed to kill bacteria by interfering with the integrity of the cytoplasmic membrane thereby causing leakage of the cytoplasmic content and cell de-energisation^39^. Based on investigations on model membrane systems, diverse modes of peptide-triggered membrane permeabilization have been suggested ranging from the formation of discrete pores and detergent-like activity to more subtle mechanisms such as perturbation of lipid packing^39^,^40^,^41^. The ability to trigger membrane rigidification combined with lipid domain formation, rather than membrane permeabilization or depolarisation, clearly distinguishes cWFW from other cationic AMPs. Hence, the case of cWFW demonstrates that cell permeabilization and de-energisation are not the only mechanisms by which membrane targeting AMPs can elicit strong bactericidal activities. The observed cross-interference with the cell wall synthetic machinery is, however, not unique for cWFW. In fact, most membrane-targeting AMPs induce cellular stress responses linked to inhibition of cell wall synthesis^42^,^43^. Based on work carried out with human β-defensin 3, Sahl and co-workers postulated a central role for lipid II and its biosynthesis pathway in the inhibition of cell wall synthesis by antimicrobial peptides^42^,^44^. Later, the linear cationic peptide MP169 was shown to trigger membrane dissociation of lipid II synthesis protein MurG as part of its complex mode of action^13^. Very recently, daptomycin was shown to target the cell wall synthesis by a more specific mechanism which includes intercalation into lipid microdomains associated with the cell wall synthetic machinery^22^. The ability of the fluidity-reducing cWFW to efficiently inhibit cell wall synthesis adds to the mounting evidence that membrane-associated steps of cell wall precursor biosynthesis are extraordinarily sensitive to membrane disturbances. The dual impairment of both the cytoplasmic membrane and cell wall synthesis therefore represents a cellular weak point which is frequently exploited by membrane targeting AMPs. It is tempting to speculate that this phenomenon contributes to the low incidence of resistant mutations emerging against cWFW (unpublished data), and in general against membrane targeting AMPs^45^.

In the context of lipid domains caused by AMPs, the discussion has focussed on the separation of anionic and zwitterionic lipids caused by preferential binding of cationic peptides to the negatively charged lipids^40^,^46^. The resulting phase boundary effects are considered the basis for the enhanced permeability of the lipid matrix^47^,^48^. In this manuscript, we provide direct microscopic *in vivo* evidence for peptide-induced formation of large lateral lipid domains in *B. subtilis*. Instead of demixing of anionic and zwitterionic lipids, however, the observed lipid domains display a difference in membrane fluidity. The cWFW-triggered domain formation is thus conceptually comparable to a phase-behaviour of lipid bilayers, and bears an intriguing similarity to cholesterol-induced membrane rigidification and lipid-raft formation^49^. The co-occurring segregation of membrane proteins adds a significant level of complexity to the phenomenon. Rather than being driven by a *bona fide* phase separation between fluid and less fluid lipid regions, the observed domain formation could also be caused by cWFW-induced changes in bilayer thickness, an effect frequently observed for antimicrobial peptides^50^. The segregation of integral membrane proteins away from the cWFW-enriched domains could be triggered by a hydrophobic mismatch between the transmembrane domains and the peptide-enriched membrane areas. The membrane areas with low density of integral membrane proteins could, in turn, attract peripheral membrane proteins and lipid dyes due to the increased surface area available for binding. Substantial amount of future work, both *in vivo* and *in vitro*, is required to understand the detailed molecular characteristics of the cWFW-triggered lipid domains. Nevertheless, our findings confirm that lipid phase separation and related protein segregation into the separate membrane domains are the key events in the mode of action of the cyclic R-, W-rich hexapeptide cWFW.

In summary, the antimicrobial mechanism of this membrane-active, non-depolarising peptide is based on substantial disruption of the lipid and protein organisation of the cytoplasmic membrane which has severe consequences for essential cellular processes as exemplified by the inhibition of cell wall synthesis. We consider the phenomenon a novel antimicrobial mode of action which provides valuable insight into the diverse mechanisms by which membrane-active peptides unfold their antimicrobial potential.

## Methods

### Peptide synthesis

As reported previously, the parent peptide cWFW was prepared by multiple solid-phase synthesis using an Fmoc/tBu strategy according to SHEPPARD^51^,^52^,^53^. Peptide purification and analysis were accomplished with high performance liquid chromatography (HPLC) on a Jasco LC-2000Plus (Japan) and Dionex UltiMate 3000 with ProntoSil 300–5-C18-H columns (250 × 4.6 mm, 5 μm) (Bischoff Chromatography, USA). Peptide mass was determined by UPLC-MS (ultra-performance liquid chromatography mass spectrometry) on an ACQUITY UPLC^®^ System (Waters) using an Ascentis^®^ Express Peptide ES-C18 column (3 × 2.1 mm, 2.7 μm) (Sigma-Aldrich). Final peptide purity was determined to be >95%. The labelled peptide derivative (FL-cWFW) containing 3-N-(7-nitrobenz-2-oxa-1,3-diazole-4-yl)-2,3-diamino-propionic acid (Dap-NBD) was purchased from Biosyntan GmbH, Berlin.

### Bacterial strains and growth conditions

Cells were inoculated from an overnight culture 1:100 and grown to mid-log phase (OD_600_ = 0.2–0.4). Unless stated otherwise, all experiments were carried out in LB medium at 37 °C. Cell numbers were determined using a Petroff-Hausser counting chamber: OD_600_ = 1 corresponds to 8.8 × 10^7^ CFU/ml *B. subtilis*. Further information on strains, conditions of gene induction, and minimal inhibitory concentration are listed in Supplementary Table 4.

### Construction of strains

For the construction of a *B. subtilis* strain expressing WALP23-GFP, the synthetic sequence encoding WALP23^54^ was constructed by annealing oligonucleotides WALP23-for and WALP23-rev, followed by PCR-filling the single-stranded regions. The resulting DNA-fragment was amplified using oligonucleotides WALP23-IFfor and WALP23-IFrev, and fused with plasmid pSG1154^55^ PCR-linearised with oligonucleotides pSG1154-for and pSG154-rev using In-Fusion Cloning (Clontech). The final plasmid was integrated into *amyE*-locus of *B. subtilis* resulting in strain HS64. For the construction of a *B. subtili*s strain expressing GFP fused with JunLZ-dimerisation domain^56^ and a membrane-targeting sequence (MTS) of *B. subtili*s MinD^57^, junLZ-MinD_MTS_ was PCR-amplified from a previously constructed *E. coli* plasmid pHJS100^57^ using oligonucleotides JunLZ-for and MTS-rev, followed by ligation into *Bam*HI/*Hind*III-linearised *E. coli*-*B. subtilis* shuttle vector pSG1729^55^. At last, the plasmid was integrated into *amyE*-locus of *B. subtilis* resulting in strain HS65. Transformation of *B. subtilis* 168 with either plasmid DNA or chromosomal DNA from donor strains was carried out as described in Hamoen *et al*.^58^. See Supplementary Table 5 for oligonucleotide sequences.

### Antimicrobial activity

The minimal inhibitory concentration (MIC), which is defined as the lowest concentration able to inhibit growth of a microorganism *in vitro*^59^, was analysed as described before^60^. Briefly, bacteria were grown to mid-log phase and diluted in growth medium to give a final cell number of 5 × 10^5^ cells/well in 96 well microtiter plates. A microdilution technique was applied where small volumes of peptide solutions were added in a serial dilution ranging from 100 to 0.05 μM. Cells were cultivated for 18 hours, 37 °C, 180 rpm, followed by photometrical detection of the optical density using a microplate reader (Tecan). Peptide concentrations were tested in triplicates in three independent experiments.

### Proteome analysis

*B. subtilis* 168/DSM402 was grown in chemically defined Belitzky minimal medium (BMM)^61^. Cultures were incubated at 37 °C under steady agitation. Minimal inhibitory concentrations (MIC) were determined as described previously^62^. In growth experiments, exponentially growing cultures were exposed to a concentration range of peptide (Supplementary Figure 1)^62^. For physiological stress experiments, concentrations were chosen that led to 50% reduction of the growth rate. For proteomic profiling, as well as for all follow up experiments performed under the same growth conditions, 8 μM cWFW were used.

Radioactive labelling of newly synthesised proteins and subsequent separation of the cytosolic proteome by two-dimensional polyacrylamide gel electrophoresis (2D-PAGE) was performed as previously described^62^. Analytical gel images were analysed as described by Raatschen & Bandow^63^ using Decodon Delta 2D 4.1 image analysis software. Proteins more than two-fold upregulated in three independent biological replicates were defined as marker proteins. Protein spots were identified by MALDI-ToF/ToF or nanoUPLC-ESI-MS/MS using a Synapt G2-S HDMS mass spectrometer equipped with a lock spray source for electrospray ionisation and a ToF detector (Waters) as previously reported (Supplementary Tables 2 and 3)^62^.

### Membrane potential measurement

Peptide-induced changes in membrane potential were measured using the voltage-sensitive fluorescent dye DiSC_3_(5). In aqueous solution DiSC_3_(5) emits strong fluorescence which is quenched upon accumulation in polarised cells. Upon depolarisation, DiSC_3_(5) is released from the cell resulting in an increased fluorescence due to de-quenching. *B. subtilis* 168 was grown at 37 °C to mid-log phase and diluted in LB medium to OD_600_ 0.2. After centrifugation for 1 min at 16,500 rpm, the supernatant was removed and cells carefully resuspended in 1 ml fresh pre-warmed LB containing 0.1 mg/ml BSA. Subsequently, 140 μl aliquots of the cell suspension were transferred into a 96 well microtiter plate and allowed to settle. 10 μl of 15 μM DiSC_3_(5) in LB/15% DMSO were added to the wells to give a final DiSC_3_(5) concentration of 1 μM. Cellular accumulation (quenching) of DiSC_3_(5) was monitored until the signal had reached stable fluorescence levels. The peptides were added at desired concentrations at 5 μl/well, and the resulting changes in DiSC_3_(5) fluorescence were analysed for at least 20 min using a Fluostar Optima, BMG Labtech. Solutions, plates and instruments were warmed to 37 °C prior to use.

### ATP measurement

For the determination of cellular ATP levels samples were incubated with increasing concentration of cWFW for 20 min, followed by flash-freeze in liquid N_2_ in order to stop metabolism. Cell lysis and measurement of cell ATP levels were carried out using ATP bioluminescence Assay Kit HSII (Roche Applied Science) following manufacturer’s instructions using a BMG Fluostar Optima luminometer. The measured luminescence was calibrated with a serial dilution of ATP and triplicate samples were equalised against the optical density of the corresponding cell suspensions.

### Ion analysis

Only metal-free plastic ware and ultrapure water (Bernd Kraft) were used. All centrifugation steps were reduced to 2 min to reduce sample handling time. Cultures were adjusted to OD_500_ = 0.4 prior to antibiotic exposure to ensure equal cell count in each sample. Samples were prepared as described by Wenzel *et al*.^13^. Briefly, *B. subtilis* 168/DSM402 was grown in BMM until early exponential growth phase. For determination of ion concentrations, subcultures were incubated with 8 μM cWFW for 15 min, followed by cell harvest by centrifugation, washed twice in TE buffer (100 mM Tris-HCl, 1 mM EDTA, pH 7.5) and once in the same buffer without EDTA. The obtained cell pellets were digested in 65% nitric acid (Bernd Kraft) at 80 °C for 16 h. Prior to element analysis, samples were diluted with ultrapure water to a final nitric acid concentration of 10%. Element concentrations were determined by inductively-coupled plasma atomic emission spectroscopy using an iCAP* 6300 Duo View ICP Spectrometer (Thermo Fisher Scientific) as described before^13^. Liquid calibration standards ranging from 10 μg/l to 10 mg/l of each element of interest (Fe, K, Mg, Mn, Na, S, P and Zn) (Bernd Kraft) were run before each series of measurements and selected standards were additionally run every 20 samples as quality control. Sulfur and phosphorus served as additional internal controls for cell mass. Element concentrations were converted into intracellular ion concentrations based on the *B. subtilis* cellular volume, which was taken as 3 × 10^–9^ μl based on average rod size determined by cryo-electron microscopy images by Matias and Beveridge^64^,^65^.

### Membrane conductivity measurements

Free-standing planar lipid membranes were formed according to an established protocol^66^ from a 20 mg/mL solution of *E. coli* lipid (Avanti Polar Lipids, Alabaster, USA) in n-decane and spread across a circular aperture (Ø 150 μm) in a polysulfone bilayer cup between two aqueous phases in a bilayer chamber (both Warner Instruments, LLC, Hamden, USA). Transmembrane current was measured with Ag/AgCl electrodes and an Axon GeneClamp 500 amplifier (Molecular Devices, Sunnyvale, USA) under voltage-clamp conditions. A 4-pole Bessel with a 3-dB corner frequency of 500 Hz was used as recording filter. The amplified signal was digitised by a PCI 6025E computer board (National Instruments, Munich, Germany) and analysed with WinEDR (Strathclyde Electrophysiology Software, Strathclyde, UK). Membrane conductivity was calculated for zero-voltage from voltage-current dependencies of the steady-state current (recorded by application of ramp voltage from −100 to 100 mV). Peptides were added at both sides of the membrane (cWFW: 10 μM, KLA-1: 0.3 μM; buffer: 10 mM HEPES, 150 mM KCl, pH 7.4, 21–23 °C). Gaussian filters of 3–11 Hz were applied to reduce noise while data processing.

### Membrane fluidity measurements

Membrane fluidity was investigated with the small hydrophobic fluorescent dye laurdan, which integrates into cellular membranes and detects changes related to the lipid packing of the surrounding bilayer^67^,^68^. The resulting changes in the fluorescence emission spectrum can be detected either spectroscopically or with fluorescence microscopy. A mathematical quantification of the emission shift is achieved by calculation of the laurdan GP (generalised polarisation) with GP = (I_435_ − I_490_)⁄(I_435_ + I_490_).

*B. subtilis* 168 cells were grown to OD_600_ ~ 0.5 in LB/0.1% glucose and incubated with 10 μM laurdan for 5 min, shaking in the dark. Subsequently, cells were washed 4 times in PBS/0.1% glucose, and 150 μl aliquots were transferred to a 96 well microtiter plate. The peptide was added at the desired final concentration at a maximum volume of 3 μl/well. The supernatant removed from the cells after the last washing step served as background for laurdan fluorescence not associated with cells. The solvent benzyl alcohol (BA) has a fluidising effect on lipid bilayers and was used as positive control (50 mM). All samples were shaken briefly before fluorescence detection at 435 ± 5 nm and 490 ± 5 nm (upon excitation at 350 ± 10 nm) on a plate reader (Fluostar Optima, BMG Labtech). Laurdan fluorescence was monitored for at least 20 min. Peptide influence on membrane fluidity was investigated in three independent experiments. Laurdan fluorescence in liposomes was recorded using an LS 50B spectrofluorometer (Perkin-Elmer Corp., Germany). Samples of 0.25 mM LUV suspension were continuously stirred during the experiment and temperature control was achieved by a built-in Peltier element. Laurdan fluorescence was detected separately at 435 and 500 nm, respectively. The average of three measurements for each preparation was calculated to determine corresponding laurdan GP values. See Scheinpflug *et al*.^69^ for detailed protocols and discussion of the laurdan-based fluidity measurements.

### Liposome preparation

For *in vitro* analysis of membrane fluidity 1-palmitoyl-2-oleoyl-sn-glycero-3-phosphoethanolamine (POPE), cardiolipin (CL, bovine brain), 1-palmitoyl-2-oleoyl-sn-glycero-3[phosphor-rac-(1-glycerol)] (POPG) and natural *E. coli* polar lipid extract were purchased from Avanti^®^ Polar Lipids (Alabama, USA). Lipid powders were dissolved in chloroform/methanol (3/1) and individual components of binary mixtures were mixed in desired combinations: POPE/CL 87.5/12.5, POPE/PG 75/25. *E. coli* lipid extract was used as obtained. 0.2 mg/ml laurdan solution in chloroform was added to each lipid mixture giving a laurdan-to-lipid molar ratio of 1.5:1000. Large unilamellar vesicles (LUVs) were prepared using a standard protocol^5^ by extrusion of lipid solution in phosphate buffer through two stacked polycarbonate filters (100 nm pore size) using a MiniExtruder (Avestin Europe, Germany). Vesicle size was ~110 nm, as determined by dynamic light scattering performed on a Zetasizer Nano ZS ZEN 3600 device (Malvern Instruments, UK). Aliquots were stored at −20 °C under argon atmosphere for replicate experiments. 1 mM lipid stock solutions were used for plain vesicles, while peptide-containing LUVs were prepared from 2 mM lipid stocks mixed with an equal volume of cWFW in phosphate buffer. Peptide concentration varied from 10 to 50 μM, corresponding to molar peptide-lipid ratios of P/L = 0.01 to 0.05. To obtain homogeneous peptide distribution the suspensions were vigorously vortexed and subjected to 1–2 cycles (5 min/cycle) in an ultrasonic bath. Vesicles were further extruded as described above. LUV size was 110–130 nm, depending on peptide-lipid molar ratio.

### Fluorescence microscopy

Peptide-induced formation of lipid domains was visualized with the membrane dye nile red. The fluorescence of the dye is very low in polar solvents but strongly increases in hydrophobic environments. Nile red intensity is considered to be independent of the interaction with certain lipid head groups but is affected by the degree of membrane fluidity^25^. Cells were grown to mid-log phase, adjusted to OD 0.2 in growth medium and incubated with 12 μM peptide for 20 min, shaking. Microscope slides were covered with a thin layer of H_2_O/1.2% agarose and transferred to 8 °C for polymerisation (adjusted to room temperature prior to use). Nile red (final concentration of 1 μg/ml) was added to the cells immediately before imaging. Fluorescence microscopy was performed using Nikon Eclipse Ti (Nikon Plan Fluor 100x/1.30 oil ph3 DLL objective).

Peptide-induced protein delocalisation was investigated with *B. subtilis* strains expressing fluorescent fusion-proteins (see supplementary Table 4). For this aim, the cells were cultivated at 37 °C to early/mid-log phase, adjusted to OD_600_ of 0.2 in growth medium, and incubated with 12 μM cWFW for 20 min at 37 °C under shaking. Defects in cell wall synthesis were analysed after 15 min pre-incubation of *B. subtilis* wild type cells with 12 μM cWFW followed by addition of 0.5 μg/ml BODIPY-labelled vancomycin (FL-Van, Thermo Fisher Scientific) mixed with an equal concentration of unlabelled vancomycin for 5 min. Fluorescence imaging was performed as described above. All microscopy experiments were carried in biological triplicates.

### Fatty acid analysis

The fatty acid composition of *B. subtilis* membranes was determined for cells grown at 37 °C in LB medium and for cells challenged with cWFW. Cell samples were withdrawn at comparable optical densities and upon re-initiation of growth of the cWFW-stressed cells in order to provide sufficient time for adaptation. The analysis was performed by conversion of lipids into fatty acid methyl esters (FAME) followed by gas chromatography. The analysis was carried out by the Identification Service of the DSMZ, Braunschweig, Germany.

## Additional Information

**How to cite this article:** Scheinpflug, K. *et al*. Antimicrobial peptide cWFW kills by combining lipid phase separation with autolysis. *Sci. Rep.* **7**, 44332; doi: 10.1038/srep44332 (2017).

**Publisher's note:** Springer Nature remains neutral with regard to jurisdictional claims in published maps and institutional affiliations.

## Supplementary Material

Supplementary Information

Supplementary Movie 1

## Figures and Tables

**Table 1:** Marker proteins upregulated upon cWFW treatment.

protein	induction factor	known or predicted function	regulon	functional category
YceC*	9.9	similar to tellurium resistance protein	SigW, SigM, SigB, SigX	cell envelope general stress
FosB	8.3	bacillithiol-S-transferase, fosfomycin resistance	SigW	cell envelope
YfhM	5.8	similar to epoxide hydrolase, general stress protein	SigW, SigB	cell envelope general stress
YthP	5.9	similar to ABC transporter (ATP-binding protein)	SigW	cell envelope
YvlB	9.0	unknown	SigW	cell envelope
GtaB	3.2	UTP-glucose-1-phosphate uridylyltransferase, general stress protein	SigB	cell envelope general stress
**LiaH**^**#**^	16.6	similar to phage shock protein	LiaRS	cell wall
RacX	12.0	amino acid racemase	SigW	cell wall
**YpuA**^**#**^	4.0	unknown	SigM	cell wall
DltA	4.2	D-alanyl-D-alanine carrier protein ligase, antimicrobial peptide resistance	SigD, SigM, SigX, Spo0A	cell wall
**PspA°**	9.5	phage shock protein A homologue	SigW, AbrV	membrane
AcsA	3.7	acetyl-CoA synthetase	CcpA, CodY	membrane
YjdA	3.4	similar to 3-oxoacyl-acyl-carrier protein reductase	unknown	membrane
YoxD	7.1	similar to 3-oxoacyl-acyl-carrier protein reductase	unknown	membrane
IspH	19.0	4-hydroxy-3-methylbut-2-enyl diphosphate reductase, isoprene biosynthesis	unknown	membrane
**NadE°**	11.0	NAD synthase	SigB	energy
NfrA	5.7	FMN-containing NADPH-linked nitro/flavin reductase	SigD, Spo0A, Spx	energy
YwrO	4.3	similar to NAD(P)H oxidoreductase	unknown	energy
CitZ	3.5	citrate synthase	CcpA, CcpC	energy
YqkF	7.7	similar to oxidoreductase	unknown	energy
TpiA	5.8	triose phosphate isomerase, glycolytic/gluconeogenic enzyme	CggR	energy
YwjI	6.4	class II fructose-1,6-bisphosphatase, gluconeogenesis	unknown	energy
FbaA	2.9	fructose 1,6-bisphosphate aldolase, glycolytic/gluconeogenic enzyme	unknown	energy
PepF	3.6	oligoendopeptidase	unknown	phosphorelay
PurH	3.4	phosphoribosylaminoimidazole carboxamide formyltransferase	PurR, G-box	nucleotide biosynthesis
GsiB	8.4	general stress protein	SigB	general stress
YvyD	6.2	general stress protein, required for ribosome dimerization in stationary phase	SigB, SigH	general stress
YdaG	5.6	general stress protein	SigB	general stress
OhrB	4.4	general stress protein	SigB	general stress
Dps	5.2	general stress protein, iron storage protein	SigB	general stress iron metabolism
ClpP	4.8	ATP-dependent Clp protease proteolytic subunit (class III heat-shock protein)	SigB, CtsR	general stress heat shock
ClpC	5.4	ATPase subunit of the ATP-dependent ClpC-ClpP protease	SigB, SigF, CtsR	general stress heat shock
GroEL	3.9	chaperonin	HrcA	chaperone heat shock
SodA	3.0	superoxide dismutase	SigB	oxidative stress
Tpx	4.1	thiol peroxidase	Spx	oxidative stress
YqiW	2.4	unknown, disulfide isomerase family	unknown	oxidative stress
YdbD	19.4	similar to manganese-containing catalase	SigB	oxidative stress
LuxS	3.4	S-ribosylhomocysteine lyase, swarming, biofilm formation, methionine salvage	unknown	motility, biofilm
MinD	3.1	cell division site placement	SigH, SigM	cell division envelope stress
SpoVG	2.4	negative effector of asymmetric septation	SigH. SinR, AbrB	cell division sporulation
YkaA	4.6	unknown	Spo0A	sporulation
SalA	3.5	negative regulator of *scoC* expression, derepression of subtilisin	ScoC, SalA	gene regulation proteolysis

*Specific marker for cell envelope stress, °specific marker for membrane stress, ^#^specific marker for inhibition of membrane-bound cell wall biosynthesis steps^43^.
