# Supplementary material for: Antimicrobial peptide cWFW kills by combining lipid phase separation with autolysis
Source: Sci Rep. 2017 Mar 9;7:44332. doi: 10.1038/srep44332 (PMC5343580; doi:10.1038/srep44332)
Supplement: Supplementary Information [file srep44332-s1.pdf]

Antimicrobial peptide cWFW kills by combining lipid phase separation with autolysis

Supplementary Information

Kathi Scheinpflug, Michaela Wenzel, Oxana Krylova, Julia E. Bandow, Margitta Dathe, and Henrik Strahl

Content:

Supplementary Figures 1-3
Supplementary Tables 1-5
Supplementary Movie 1
Supplementary References

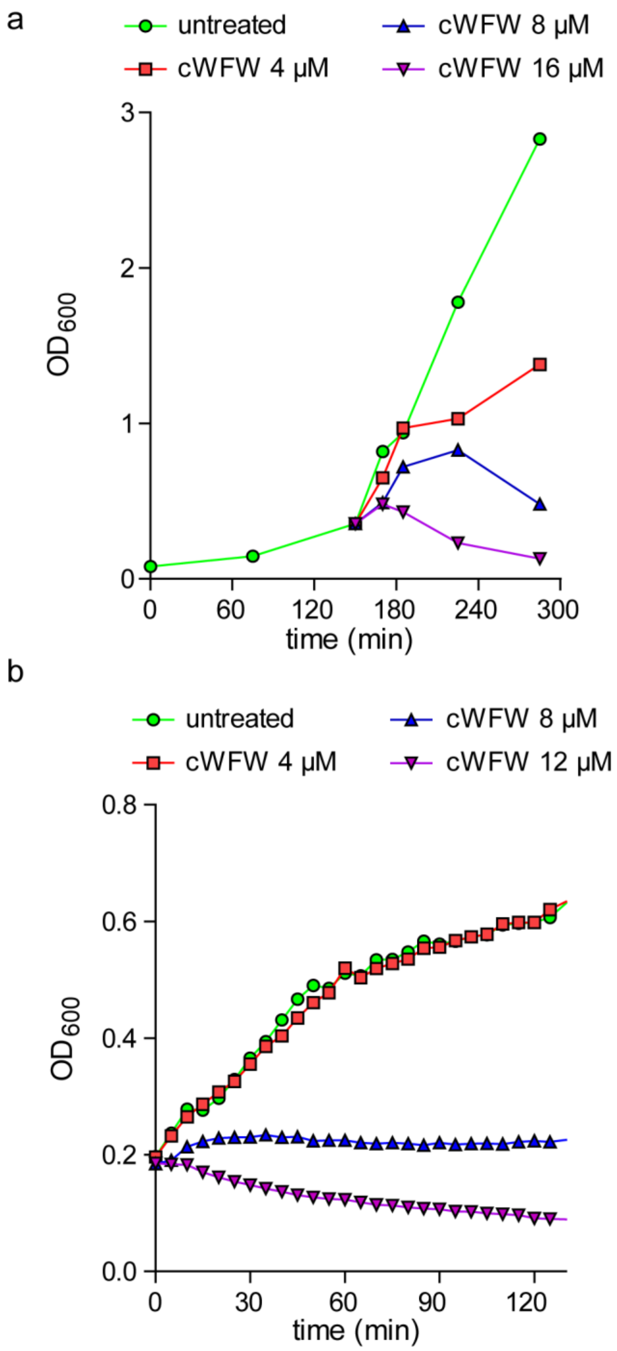

Supplementary Fig. 1: Concentration-dependent growth inhibition of *B. subtilis* in the different growth media used in this study

(a) Growth curves of *B. subtilis* 168/DSM402 at 37 °C in BMM upon addition of different concentrations of cWFW. The time point of cWFW addition is indicated with a dashed line. The selected inhibitory concentration (8 µM) was used for proteome and ion content analysis. (b) Growth curves of *B. subtilis* 168 at 37 °C in LB-medium in the presence of different

concentrations of cWFW as measured on microtiter plates. The growth inhibition upon peptide addition to a logarithmically growing culture at OD_{600}=0.2 is shown. The graphs depict a representative of three independent measurements.

a

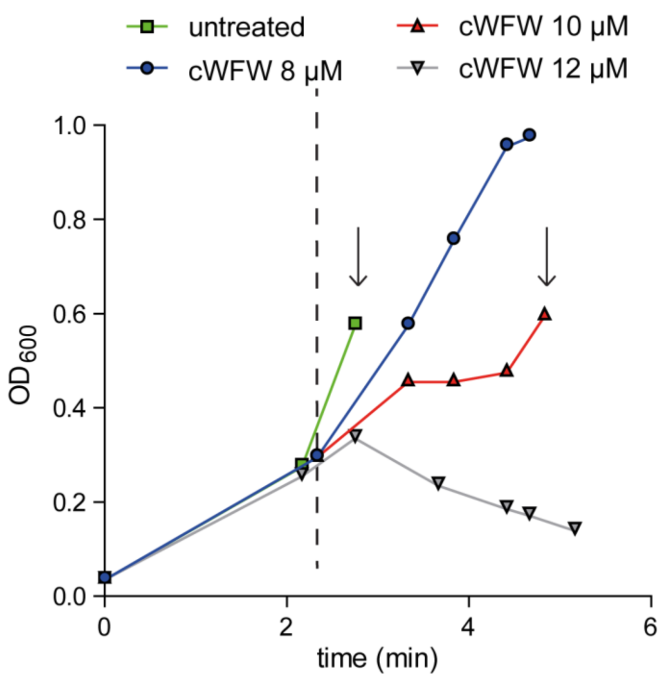

b

	untreated	10 µM cWFW (2.5h)
$C_{14:0}$ iso	1.5	2.0
$C_{14:0}$	0.5	0.55
$C_{15:0}$ iso	18.0	23.8
$C_{15:0}$ anteiso	37.1	37.3
$C_{16:0}$ iso	5.4	5.8
$C_{16:0}$	4.9	4.7
$C_{17:0}$ iso	15.0	13.1
$C_{17:0}$ anteiso	16.0	11.3
$C_{18:0}$	1.2	0.8
sum	99.4	99.4
saturated	6.5	6.1
iso	39.8	44.8
anteiso	53.1	48.6
iso/anteiso	**0.75**	**0.92**
short ($<C_{16}$)	57.0	63.6
long ($\geq C_{16}$)	37.0	30.0
short/long	**1.54**	**2.12**

Supplementary Fig. 2: Membrane fatty acid adaptation in response to cWFW

(a) Growth curves of *B. subtilis* 168 at 37 °C in LB medium upon addition of different concentrations of cWFW. The time points of cell harvest for fatty acid analysis are indicated with arrows. The graphs depict a representative of two independent measurements. (b) The cellular fatty acid profile in the absence of cWFW and after 2.5 h incubation with 10 µM cWFW. Strain used: *B. subtilis* 168 (wild type).

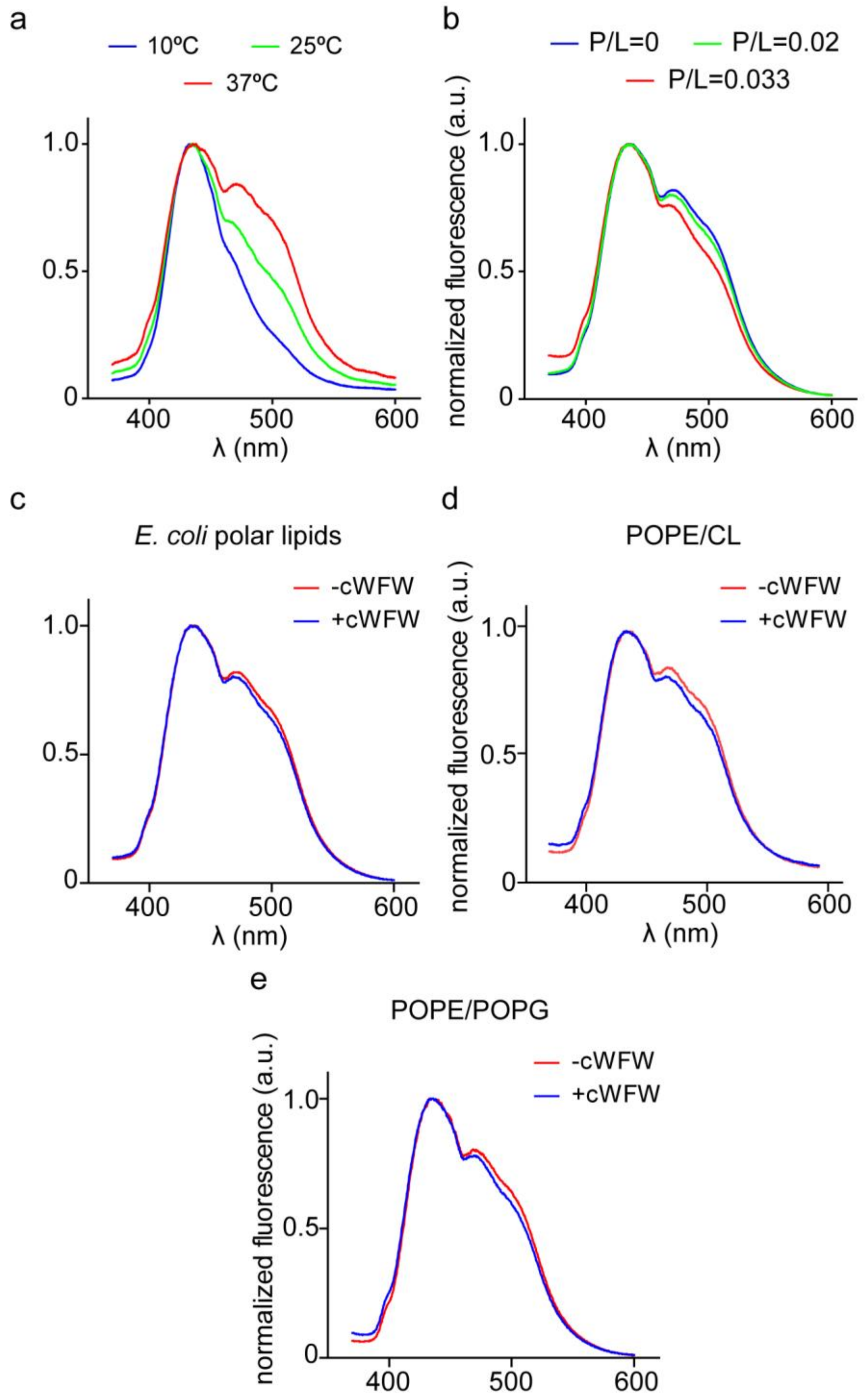

Supplementary Fig. 3: cWFW-dependent laurdan fluorescence emission spectra in LUVs of varying lipid composition

(a) Fluorescence emission spectrum of laurdan incorporated into large unilamellar vesicles (LUVs) formed of *E. coli* polar lipid extract at different temperatures. Note the spectral shift caused by reduced membrane fluidity at lower temperatures. (b) Fluorescence emission spectrum of laurdan stained LUVs formed of *E. coli* polar lipids at 37 °C in the presence of

different concentrations of cWFW. P/L indicates peptide-to-lipid molar ratio. (C-D) Fluorescence emission spectrum of laurdan stained LUVs formed of *E. coli* polar lipids, a binary mixture of POPE and cardiolipin (POPE/CL) and a binary mixture of POPE and POPG, respectively, in the absence and presence of cWFW (P/L=0.02). The spectra depict a representative of two independent measurements.

Supplementary Table 1: Comparison of cWFW with proteome response library

protein	cWFW	MP196	vancomycin	mersacidin	gallidermin	nisin	gramicidin S	valinomycin	gramicidin A	ionomycin	triton X-100
**YceC***	x	x	x	x	x		x			x	x
FosB	x	x									
YfhM	x	x								x	x
YthP	x	x									
YvlB	x	x			x		x	x		x	x
**LiaH#**	x	x		x	x	x	x				
RacX	x	x					x	x			
**YpuA#**	x			x	x	x	x				
DltA	x	x			x						
**PspA°**	x	x			x	x	x	x	x		x
YjdA	x	x					x	x			
YoxD	x	x	x				x		x		
**NadE°**	x	x			x	x	x	x	x		x
NfrA	x	x			x		x	x			
YwrO	x	x					x				x
**CitZ**	x	x						x		x	
GsiB	x	x					x	x			
YdaG	x	x									
Dps	x	x						x			
ClpP	x						x				
Tpx	x										x
YqiW	x									x	
YdbD	x	x					x				
SpoVG	x	x						x			x
**total markers**	**52**	36	5	13	25	8	21	22	5	38	26

*specific marker for cell envelope stress, °specific marker for membrane stress, #specific marker for inhibition of membrane-bound cell wall biosynthesis steps [1].

Supplementary Table 2: Details of mass spectrometric protein spot identification (nLC-ESI-MS)

protein	mass weight [Da]	pI	PGLS score	number of matching proteins	coverage [%]	number of peptides with fragment data	precursor mass error [ppm]	product mass error [ppm]
**AcsA**	64850	5.54	4431	48	36	48	1.38	4.08
**ClpC**	90063	5.75	22605	182	60	159	1.55	4.88
**FosB**	17161	6.14	754	4	17	4	1.57	2.17
**NfrA**	28302	5.73	9602	24	33	20	1.58	4.45
**SalA**	38614	5.23	6829	18	44	33	1.42	3.67
**YfhM**	32737	6.07	7499	22	46	20	1.23	3.88
**YpuA**	31275	4.51	20679	38	53	30	1.75	4.39
**GroEL**	57388	4.53	35524	178	90	178	2.80	7.77
**OhrB**	14591	4.73	7866	23	94	23	2.07	9.24
**GtaB**	33049	4.91	36499	111	96	111	2.18	7.66
**TpiA**	27012	4.79	1709	10	32	10	2.21	10.05
**PepF**	77024	5.49	15612	116	63	116	1.63	7.80
**PurH**	55719	5.13	4098	60	51	60	3.24	8.39
**YwjI**	33929	5.06	15972	43	74	43	2.01	7.46
**FbaA**	30381	5.02	12444	63	85	63	2.06	8.03
**MinD**	29388	4.98	16778	43	62	43	1.68	7.57
**YkaA**	23829	4.90	7090	27	63	27	1.86	9.71

Supplementary Table 3: Details of mass spectrometric protein spot identification (MALDI-ToF/ToF)

protein	mass weight [Da]	pI	mascot protein score	mascot protein score % C.I.	number of peptides used for identification
**CitZ**	41702	5.55	444	100	16
**ClpP**	21668	5.19	353	100	12
**DltA**	55773	5.10	276	100	13
**Dps**	16583	4.64	168	100	8
**GlpK**	55045	5.16	96	100	11
**GroEL**	57388	4.53	146	100	11
**GsiB**	13789	5.31	122	100	5
**IspH**	34902	5.68	91	100	2
**LiaH**	25682	6.20	112	100	13
**LuxS**	17703	5.29	301	100	5
**NadE**	30376	5.07	459	100	16
**PspA**	25125	5.87	91	100	11
**RacX**	25270	5.46	73	100	3
**SodA**	22561	5.33	414	100	8
**SpoVG**	10886	5.25	348	100	10
**Tpx**	18204	4.89	403	100	15
**YceC**	21810	5.46	224	100	14
**YdaG**	15867	5.33	111	100	5
**YdbD**	30238	5.06	103	100	10
**YjdA**	27432	5.74	55	99	5
**YoxD**	25283	5.48	434	100	20
**YqiW**	16186	5.00	137	100	7
**YqkF**	34695	5.30	535	100	14
**YthP**	26490	5.39	195	100	5
**YvlB**	41056	5.50	156	100	10
**YvyD**	21966	5.36	393	100	11
**YwrO**	19942	5.33	166	100	7
**YxaB**	39738	4.72	452	100	18

Supplementary Table 4: Strains used in this study

Strain	Relevant Genotype	Induction	MIC	Source
B. subtilis 168	wild type	-	6*	2
B. subtilis 168/DSM402	wild type	-	8[#]	3
B. subtilis KS69	*amyE::spc Pxyl-msfgfp-mreB*	0.3% xyl	nd	this work
B. subtilis KS70	*amyE::spc Pxyl-msfgfp-mbl*	0.3% xyl	nd	this work
B. subtilis TNVS175	*amyE::spc Pxyl-murG-msfgfp*	0.05% xyl	nd	4
B. subtilis KS19	*lytABC::neo lytD::tet lytE::cat lytF::spc*	-		this work
B. subtilis L16648	*lytABC::neo lytD::tet lytE::cat lytF::spc*	-	nd	5
B. subtilis HB5343	*psd::ery*	-	6*	6
B. subtilis SDB206	*ywiE::neo clsB::spc clsA::ery*	-	6*	7
B. subtilis BS23	*cat atpA-gfp:Pxyl-'atpA*	0.5% xyl	nd	8
B. subtilis HS41	*amyE::tet Pxyl-yhaP-gfp*	1% xyl	nd	9
B. subtilis HS64	*amyE::spc Pxyl-WALP23-gfp*	1% xyl	nd	this work
B. subtilis KR318	*amyE::psc Phyperspank -spoVM–gfp*	1 mM IPTG	nd	10
B. subtilis HS65	*amyE::spc Pxyl-gfp-junLZ-*MTS_{MinD}	1% xyl	nd	this work
B. subtilis HS208	*amyE::spc Pxyl-*MTS_{SepF}*-junLZ-gfp*	1% xyl	nd	11

*determined in LB, [#]determined in BMM

Supplementary Table 5: Oligonucleotides

Oligonucleotide	Sequence
JunLZ-for	gcgcgGGATCCTTGGTGGTCGTATCGCTCGTCC
MTS-rev	gcgcgAAGCTTAAGATCTTACTCCGAAAAATGA
pSG1154-for	ATGAGTAAAGGAGAAGAACTTTTCAC
pSG154-rev	CATCCTAGGAATCTCCTTTCTAG
WALP23-for	ATGGCTTGGTGGTTAGCCTTGGCATTGGCGCTTGCTCTCGCCCTGGC
WALP23-rev	CGCCCACCACAGGGCTAACGCCAATGCCAGGGCGAGAGCAAGCGCC
WALP23-IFfor	GAGATTCCTAGGATGGCTTGGTGGTTAGCCTTG
WALP23-IFrev	TCTCCTTTACTCATTCCTGAGCCGCTTCCTGACGCCCACCACAGGGCTAAC

Supplementary Movie 1: cWFW-dependent cell lysis

Growth and lysis of *B. subtilis* 168 at 37 °C in solid LB medium in the absence and presence of cWFW (25 µM). Strain used: *B. subtilis* 168 (wild type).

SUPPLEMENTARY INFORMATION REFERENCES

1 Wenzel, M. *et al.* Proteomic response of *Bacillus subtilis* to lantibiotics reflects differences in interaction with the cytoplasmic membrane. *Antimicrob. Agents Chemother.* **56**, 5749-5757 (2012).

2 Barbe, V. *et al.* From a consortium sequence to a unified sequence: The *Bacillus subtilis* 168 reference genome a decade later. *Microbiology* **155**, 1758-1775 (2009).

3 Spizizen, J. Transformation of biochemically deficient strains of *Bacillus subtilis* by deoxyribonucleate. *Proc. Natl. Acad. Sci. U. S. A.* **44**, 1072-1078 (1958).

4 Müller, A. *et al.* Daptomycin inhibits cell envelope synthesis by interfering with fluid membrane microdomains. *Proc. Natl. Acad. Sci. U. S. A. In Press* (2016).

5 Margot, P. & Karamata, D. Identification of the structural genes for n-acetylmuramoyl-l-alanine amidase and its modifier in *Bacillus subtilis* 168: Inactivation of these genes by insertional mutagenesis has no effect on growth or cell separation. *Mol. Gen. Genet.* **232**, 359-366 (1992).

6 Salzberg, L. I. & Helmann, J. D. Phenotypic and transcriptomic characterization of *Bacillus subtilis* mutants with grossly altered membrane composition. *J. Bacteriol.* **190**, 7797-7807 (2008).

7 Kawai, F. *et al.* Cardiolipin domains in *Bacillus subtilis* marburg membranes. *J. Bacteriol.* **186**, 1475-1483 (2004).

8 Johnson, A. S., van Horck, S. & Lewis, P. J. Dynamic localization of membrane proteins in *Bacillus subtilis*. *Microbiology* **150**, 2815-2824 (2004).

9 Strahl, H., Bürmann, F. & Hamoen, L. W. The actin homologue MreB organizes the bacterial cell membrane. *Nat. Commun.* **5** (2014).

10 Ramamurthi, K. S., Clapham, K. R. & Losick, R. Peptide anchoring spore coat assembly to the outer forespore membrane in *Bacillus subtilis*. *Mol. Microbiol.* **62**, 1547-1557 (2006).

11 Duman, R. *et al.* Structural and genetic analyses reveal the protein SepF as a new membrane anchor for the Z ring. *Proc. Natl. Acad. Sci. U. S. A.* **110**, 4601-4610 (2013).
